# Comparative transcriptomic analysis of hypothalamus-pituitary-liver axis in bighead carp (*Hypophthalmichthys nobilis*) with differential growth rate

**DOI:** 10.1186/s12864-019-5691-4

**Published:** 2019-04-30

**Authors:** Beide Fu, Xiaomu Yu, Jingou Tong, Meixia Pang, Ying Zhou, Qingshan Liu, Wenjing Tao

**Affiliations:** 1 State Key Laboratory of Freshwater Ecology and Biotechnology, Institute of Hydrobiology, Innnovation Academy of Seed Design, Chinese Academy of Sciences, Wuhan, 430072 People’s Republic of China; 2 University of Chinese Academy of Sciences, Beijing, 100049 China; 3 Key Laboratory of Freshwater Fish Reproduction and Development (Ministry of Education), Key Laboratory of Aquatic Science of Chongqing, School of Life Sciences, Southwest University, Chongqing, 400715 China

**Keywords:** Comparative transcriptomic, Bighead carp, Hypothalamus-pituitary-liver axis, Growth rate

## Abstract

**Background:**

Growth rate is one of the most important features for aquaculture species and deciphering its regulation mechanism has great significance both in genetics and in economics. Hypothalamus-pituitary growth axis (HP growth axis) or neuro-endocrine axis plays a vital role in growth regulation in different aquaculture animals.

**Results:**

In this study, the HP and liver transcriptomes of two female groups (H and L) with phenotypically extreme growth rate were sequenced using RNA-Seq. A total of 30,524 and 22,341 genes were found expressed in the two tissues, respectively. The average expression levels for the two tissues were almost the same, but the median differed significantly. A differential expression analysis between H and L groups identified 173 and 204 differentially expressed genes (DEGs) in HP and liver tissue, respectively. Pathway analysis revealed that DEGs in HP tissue were enriched in regulation of cell proliferation and angiogenesis while in liver tissue these genes were overrepresented in sterol biosynthesis and transportation. Genomic overlapping analyses found that 4 and 5 DEGs were within growth-related QTL in HP and liver tissue respectively. A deeper analysis of these 9 genes revealed 3 genes were functionally linked to the trait of interest. The expression of 2075 lncRNAs in HP tissue and 1490 in liver tissue were also detected, and some of lncRNAs were highly expressed in the two tissues.

**Conclusions:**

Above all, the results of the present study greatly contributed to the knowledge of the regulation of growth and then assisted the design of new selection strategies for bighead carp with improved growth-related traits.

**Electronic supplementary material:**

The online version of this article (10.1186/s12864-019-5691-4) contains supplementary material, which is available to authorized users.

## Background

Growth is among the most important traits in breeding and therefore is a main target in most genetic selection programs. Hypothalamus-pituitary growth axis (HP growth axis) or neuroendocrine axis plays a vital role in growth regulation in different animals. Major genes involved in this axis are somatostatin (*SS*) and growth hormone releasing hormone (*GHRH*) which are secreted by hypothalamus, growth hormone (*GH*) synthase secreted by pituitary and insulin-like growth factor-I (*IGFI*) released by target organs, such as liver [11]. Although much work has been done to illustrate the functions about these genes in HP growth axis [23, 25], little is known regarding the whole regulation network from these genes to their direct phenotype, and growth rate in various animals. Compared with the great importance of HP growth axis in growth regulation, large-scale gene expression studies between groups with different growth rates are still too few to conclude some generalities for the main regulating genes.

Comparative transcriptome analysis has been widely used in different species to decipher the regulation of growth. In blunt snout bream, Li et al. found 92 differential expressed genes between fast-growth family and slow-growth family under normoxia in liver and gill transcriptome [16]. In swimming crab, 49 up-regulated and 68 down-regulated unigenes were discovered when comparing small size and large size crabs. GO (gene ontology) enrichment analysis showed the up-regulated genes were involved in cell communication, ovarian follicle cell development and cell division [19]. This method has also been used in other animals with the same goal, such as pig [24], cattle [20] and chicken [4]. However, these studies mainly focused on expression pattern of one specific organ or pooled organs together. To our knowledge, no large-scale transcriptome study has been reported about the expression difference in the whole HP-growth axis.

Bighead carp is an economically important freshwater fish in China, and its annual production was over 3 million tons in 2013 [8]. Recent rapid development of bighead carp farming industry caused great demand for juveniles with better growth-related traits. However, few studies had been carried out about the genetic mechanisms underlying the regulation of growth in this fish, thereby hindered the molecular breeding process for faster growing bighead carp [11]. In this study, we sequenced the hypothalamus-pituitary transcriptome as well as liver transcriptome for two full-sibling groups with different growth rates and hope to uncover differential expression genes (DEGs) in the two organs. Then through the regulation network, we aim to discover the regulating role these DEGs perform and how they cooperate to influence the growth rate.

## Results

### High throughput RNA sequencing and reads mapping

Among all female siblings from one pair of parents, we used three samples with largest body weight as H group, while three samples with smallest body weight as L group (Table 1). The body weight difference among the two groups was significant with t-test (*p* = 0.01). Two tissues in two groups (H = 3, L = 3) of phenotypically extreme for growth were sequenced using Illumina RNA sequencing. One is hypothalamus-pituitary mixed tissue (HP) and the other is liver tissue. For HP tissue, we got 28.53, 30.76, 31.77 million reads for H group and 31.60, 31.61, 30.73 for L group, respectively. For liver tissue, we obtained 26.89, 30.77, 32.21 million reads for H group and 31.71, 29.62, 30.02 million reads for L group, respectively (Table 2.). The total length of the reads was about 108.81 Gb, which was about 120.9-fold of the whole bighead carp genome (about 0.9 Gb). After filtering low-quality reads, 80.23–85.80% of the raw sequencing reads were kept as high-quality reads. In these mapped reads, 59.91–67.53% were aligned concordantly exactly one time and 15.25–19.13% were aligned concordantly more than one time (Table 2).

**Table 1:** Body weight for all 6 samples used in this study

Sample ID	First BW (Kg)	Second BW (Kg)	Third BW (Kg)	Group
2175	0.081	0.1684	0.8449	H
2087	0.0849	0.1657	0.7584	H
2262	0.0921	0.1962	0.7385	H
2179	0.0419	0.0846	0.4965	L
2236	0.0434	0.0755	0.4836	L
2216	0.036	0.0612	0.4777	L

The first BW were measured after fertilization 190 days. The second BW were measured after fertilization 361 days. The third BW were got after fertilization 550 days

*BW* Body weight

**Table 2:** Summary for RNA-SEQ information in all 12 samples

ID	Sample name	Raw reads number	Filtered Read number	Filtered Ratio (%)	Alignment rate (%)	Concordantly mapped once (%)
T01	2175liver	26,891,820	22,939,890	85.30	92.59	62.51
T02	2087liver	30,771,063	26,403,790	85.81	92.05	61.55
T03	2262liver	32,216,234	26,845,513	83.33	92.50	61.62
T04	2179liver	31,712,653	27,077,265	85.38	92.23	61.73
T05	2236liver	29,621,899	25,262,329	85.28	92.08	61.02
T06	2216liver	30,023,436	25,342,065	84.41	92.02	59.91
T07	2175HP	28,534,878	24,146,563	84.62	92.98	62.62
T08	2087HP	30,762,452	25,731,313	83.65	92.69	62.88
T09	2262HP	31,775,826	26,454,880	83.25	93.05	64.55
T10	2179HP	31,607,048	26,369,442	83.43	93.77	65.63
T11	2236HP	31,618,689	25,368,078	80.23	92.74	66.02
T12	2216HP	30,738,150	25,861,409	84.13	93.31	67.53

Concordantly mapped once rate means the paired reads were mapped to bighead carp genome only once with concordantly distance

*HP* Hypothalamus-pituitary tissue

### Gene expression analysis for the two groups

As there wasn’t an available annotation file for bighead carp genome, we had to merge all 12 GFF files produced by StringTie to reconstruct all transcripts and genes expressed in HP and liver tissues. In the combined GFF file, there were 32,348 genes and 52,076 transcripts. Based on this annotation file, we got the expression levels (FPKM, Fragments Per Kilobase of transcript per Million mapped reads) for all genes in 12 libraries.

In HP tissue, 30,524 genes were expressed in at least three samples. In these genes, 430 were highly expressed (FPKM> 100 in at least three samples) and were enriched in GnRH signaling pathway, oocyte meiosis, PPAR signaling pathway, and et al. (Additional file 3: Table S3). In detail, we found that many house-keeping genes were among highly-expressed genes, such as 75 genes from Ribosome, 46 genes from Metabolic pathways and 23 genes from Oxidative phosphorylation et al. We also found the hormone genes expressed specifically in the pituitary gland were among highly expressed genes, like growth hormone (*GH*), prolactin (*PRL*), Lutropinsubunitbeta (*LHB*) [21]. Other hypothalamus specific genes were also found expressed in HP tissue, like glial fibrillary acidic protein (*gfap*), myelin basic protein (*mbpa* and *mbpb*) and Carboxypeptidase E (*cpe*).

In liver tissue, 22,341 genes were expressed in at least three samples with FPKM> 0.1. Among these genes, we found 574 genes were highly expressed (FPKM > 100 in at least three samples, Additional file 4: Table S4). KEGG enrichment analysis indicated that the highly expressed genes were enriched (FDR < 0.05) in steroid biosynthesis, glutathione metabolism, oxidative phosphorylation, PPAR signaling pathway and other pathways involved mainly in liver. (Additional file 1: Table S1). Among the top 50 expressed genes in liver, there were 17 ribosomal protein genes (*rpl19, rpl8, rpl10a, rpl23a, rpl7, rpl11, rpl18a, rpl35, rpl21, rpl24, rpl15, rps2, rps25, rps23, rps4x, rps6, rpsa*), 4 lipoprotein (*apoa2, fabp10a, apoc1, apoa1b*), 3 complement factor genes (*fga, fgg, c3a.1*), 1 glycoprotein (*tfa*) and other genes (Additional file 2: Table S2).

**Fig. 1:**
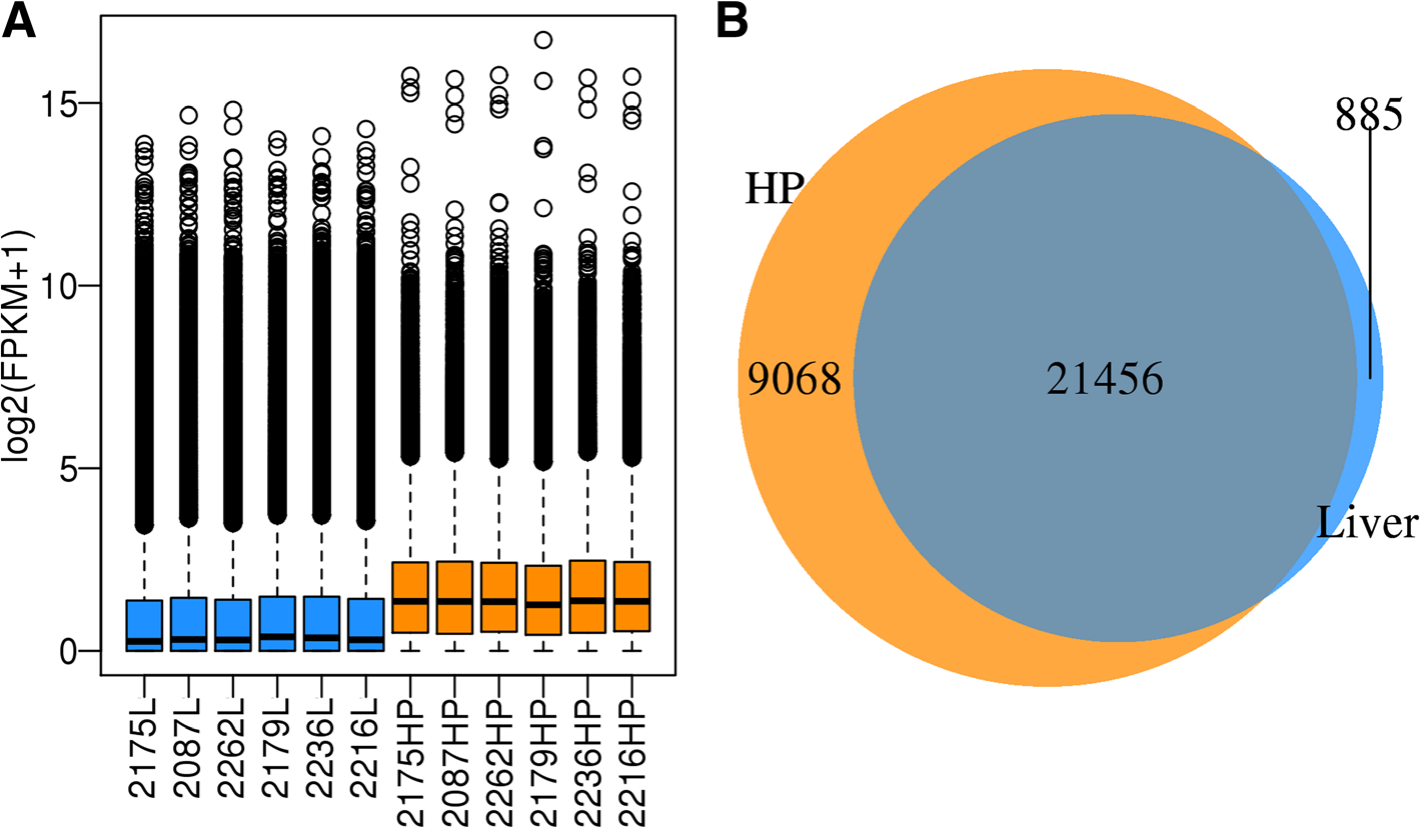
The gene expression pattern for Hypothalamus-pituitary (HP) and liver (L) tissue. **a** The blue samples are for liver tissue, and the yellow samples are for Hypothalamus-pituitary tissue. FPKM: Fragments Per Kilobase of transcript per Million mapped reads. The black bar indicated the average FPMK in the tissue. **b** Gene co-expression in the two tissues. Most of genes were co-expressed in the two tissues

**Fig. 2:**
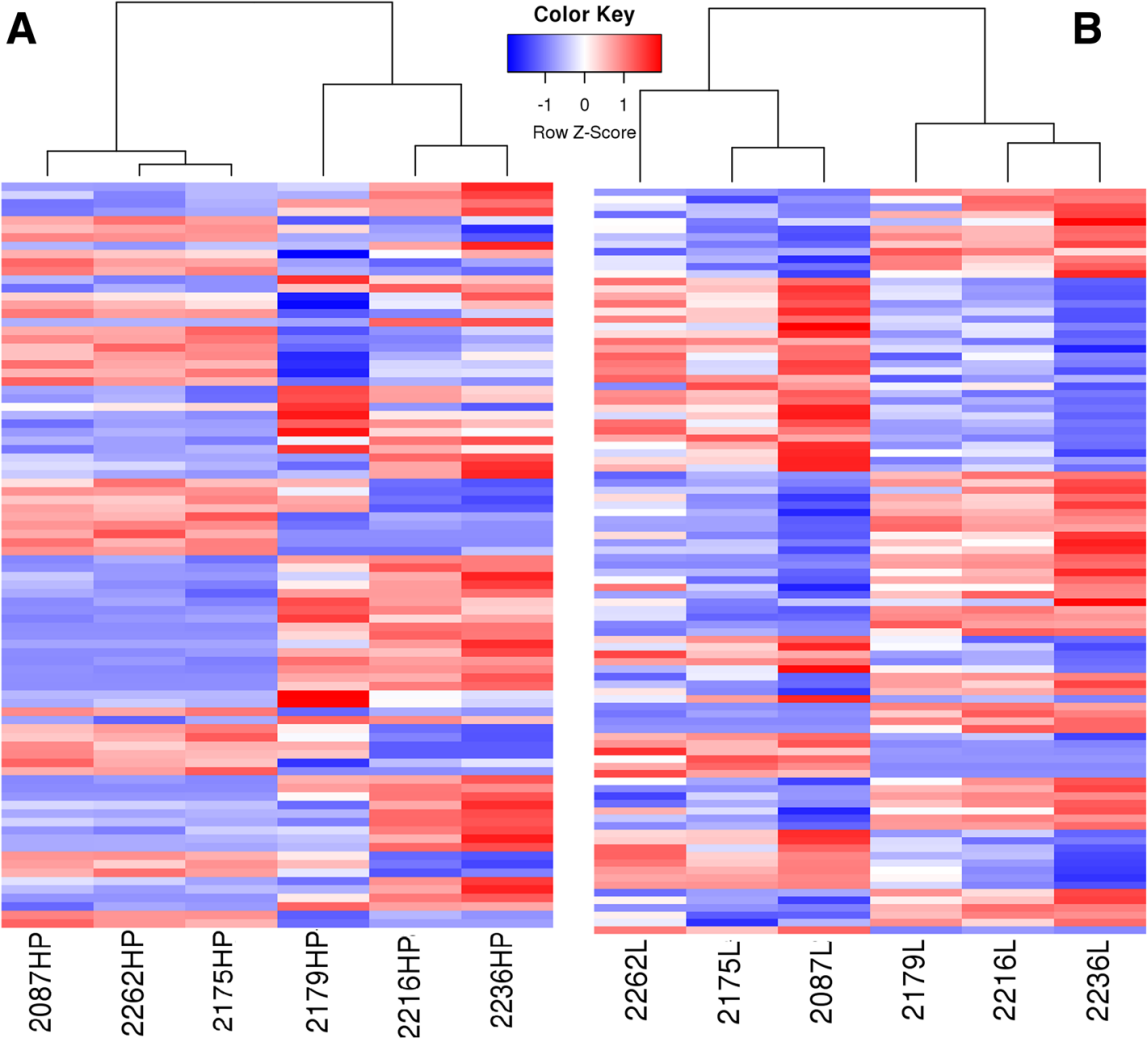
Heatmap for Hypothalamus-pituitary (HP) and liver (L) tissue. **a** Heatmap for HP tissue. **b** Heatmap for liver tissue

In the two tissues, we found 21,456 genes were co-expressed (Fig. 1b). The average expression levels for the two tissues were almost the same, but the median differed significantly (Fig. 1a and Table 3). And the clustering map showed the two groups in two tissues were clearly separated (Fig. 2). At the same time, 209 genes were highly expressed in both tissues, and these genes were enriched in several cell house-keeping KEGG pathways, like oxidative phosphorylation (18 genes out of 139, FDR = 0), ribosome (74 genes out of 134, FDR = 0), metabolic pathways (26 genes out of 1329, FDR = 1.18E-06) and protein processing in endoplasmic reticulum (10 genes out of 181, FDR = 2.47E-06). These results were consistent with the ones observed in other animal species with RNA-seq [6]. We also found that 365 liver-specific highly expressed genes which were enriched in carbon metabolism (17 genes out of 130, FDR = 0), glycolysis (12 genes out of 75, FDR = 1.06E-10) and biosynthesis of amino acid (11 genes out of 86, FDR = 1.01E-08). At the same time, 221 HP-specific highly expressed genes were found which were enriched in glycolysis (11 genes out of 75, FDR = 2.47E-10), biosynthesis of amino acids (10 genes out of 86, FDR = 9.71E-10) and phagosomes (12 genes out of 157, FDR = 9.71E-10).

**Table 3:** Quarterly expression for all 12 libraries in this study

Library Name	Min	1st Qu.	Median	Mean	3rd Qu.	Max.
FPKM.T01	0.00	0.12	1.67	26.15	6.99	19,055.06
FPKM.T02	0.00	0.13	1.80	27.06	7.42	24,824.55
FPKM.T03	0.00	0.15	1.76	27.25	6.80	27,092.80
FPKM.T04	0.00	0.23	2.01	25.75	7.14	18,796.35
FPKM.T05	0.00	0.20	1.98	26.27	7.10	17,580.54
FPKM.T06	0.00	0.15	1.73	26.91	7.18	21,565.35
FPKM.T07	0.00	3.13	6.06	25.94	12.32	55,050.21
FPKM.T08	0.00	3.16	6.20	25.89	12.77	51,713.46
FPKM.T09	0.00	3.09	6.01	27.13	12.45	55,643.91
FPKM.T10	0.00	2.84	5.62	27.01	11.68	108,529.41
FPKM.T11	0.00	3.20	6.30	25.59	13.03	52,953.14
FPKM.T12	0.00	3.14	6.12	26.41	12.76	54,059.85

The expression value comes from the FPKM for each gene in 12 libraryies. 1st Qu. Means 25% observations are below this quantity. 3rd Qu. Means 75% observations are below this quantity

### Differential gene expression analysis

After getting all the expression levels of genes in two tissues, we used Ballgown to do the differential expressed genes (DEGs) analysis. We only kept genes with variance larger than 1 in each tissue for the following analysis [18]. In HP tissue, 11,629 genes were kept and 173 genes were significantly differentially expressed. Among them, 96 genes were up-regulated in H group while the rest 77 genes showed higher expression in the L group. The expression fold change for these DEGs ranged from 1.50 to 7.81. The GO enrichment completed by GeneSCF [28] on the 173 DEGs indicated that some GO biological processes related to growth were overrepresented: regulation of cell proliferation (GO:0042127, adjusted *P* value = 0.094), fibroblast growth factor receptor signaling pathway involved in forebrain neuron fate commitment (GO:0030866, adjusted *P* value = 0.094), and angiogenesis (GO:0001525, adjusted *P* value = 0.104). Similarly, among the GO molecular function enriched in DEGs, many processed related to growth could be identified: oxidoreductase activity (GO:0016491), lipid binding (GO:0008289), cholesterol transporter activity (GO:0017127) and catalytic activity (GO:0003824).

The hypothalamus is the main gland regulating growth, food intake, and fat accumulation. To our best known, no comparative HP transcriptome between differential growth rates had been done in fish. In pig, hypothalamic transcriptome analysis was conducted between pigs with different growth rates and fatness [23]. They found 210 DEGs between two groups. In our results, we found some genes overlapped with DEGs in pig’s hypothalamic transcriptome analysis, like *rimbp2* and *SLC44A1*. In previous study, hypothalamic *LEPR* gene was found relevance in growth and fatness. Unfortunately, despite the obvious importance of this gene [22], we didn’t find this gene to be differentially expressed between the two groups in the HP tissue, which is the same as Perez-Montarelo’s results [24].

In liver tissue, 7363 genes were kept under the stringent criteria and 204 genes were found differentially expressed between H and L group. In these genes, we found that 86 genes were up-regulated in H group and 118 were down-regulated in H group. To gain insight into the biological processes that differed between groups, the list of DEGs was explored using GeneSCF [28]. At first, the bighead carp gene IDs were converted to zebrafish genes and then the results indicated that many GO biological processes related to sterol biosynthesis and transportation were over-represented: cholesterol biosynthetic process (GO:0006695), cholesterol transport (GO:0030301), sterol biosynthetic process (GO:0016126), ergosterol biosynthetic process (GO:0006696) and cholesterol metabolic process (GO:0008203).

### Overlapping with growth-related QTL in bighead carp

To identify whether the DEGs in this study were within bighead carp growth-related QTLs (quantitative trait loci), we mapped all the markers in those QTLs [10] to the reference genome and then used the location information to get DEGs that were within or nearby growth-related QTLs (less than 1 Mb in distance). In the DEGs in HP tissue, we found four genes (*slc1a7b*, *FUK*, *ABCF2* and *si:ch211-189 k9.2*) were within the growth-related QTLs. While in liver tissue, five genes (*pla1a*, *arhgap12a*, *cyp2x9*, *cog7* and one unannotated gene) were within five QTLs. In the two tissues, only one QTL located in LG19 overlapped with DEG in both tissues, but the two DEGs laid in different ends of this QTL.

### Identification of lncRNA in bighead carp

We used a method combining computational and expression data in the two tissues to identify long non-coding RNA (lncRNA) genes in bighead carp. After using the coding probability and exon number as filtering criteria for each transcript, we got 2442 candidate lncRNAs in bighead carp genome. Then we found that 1490 lncRNAs were expressed in liver and 2075 were expressed in HP tissue, among which 1326 lncRNAs were co-expressed in the two tissues. Therefore, we got 164 liver-specific expressed lncRNAs and 749 HP-specific expressed lncRNAs. Meanwhile, we found that some of the lncRNAs were highly expressed in the two tissues. In liver, 69 lncRNAs were among the top 1000 highly expressed transcripts. And in HP tissue, 68 lncRNAs were within the top 1000 transcripts with highest expression levels. Between the two groups, 38 transcripts were overlapped with each other. To understand the evolutionary history of these lncRNAs in bighead carp, we mapped all 2442 candidate lncRNAs to 4852 zebrafish lncRNAs downloaded from NONCODEV5 [7] database and found 471 lncRNAs got positive results. This phenomena might be caused by the loose evolutionary pressure on the non-coding sequences in lncRNAs [30], and this phenomena was similar to that in *Oreochromis niloticus*, which had 142 positive results out of 5634 lncRNAs.

## Discussion

Growth is a process that is controlled by the interaction of genetic background and the environment. To avoid the growth rate difference caused by different sex, we only used female samples descended from one pair of parents. All siblings were reared in the same pond in the aquaculture farm to eliminate the influence of environment. For the reads generated from the H and L groups, about 92% of them were mapped to bighead carp genome, this number was much higher than previous studies in bighead carp [11]. And we thought this result offered us a solid ground for following analysis.

Based on the good quality of sequencing reads, we utilized a widely accepted pipeline to find DEGs in the two tissues [25]. In 204 DEGs in liver tissue, we found some genes related to growth in different animals. *Acsl4a* gene was found within regulation of p38MAPK cascade, and p38 MAPK signaling pathway was downstream of IGF-I receptor [5]. *IGFALS* deficiency is a subtype of primary IGF-I deficiency and has been associated with insulin resistance, full growth potential and muscle size [13]. Besides, *IGFALS* seems to influence circulating IGFBP-3, which is the main binding protein of IGF-I [12]. In our previous study, we found that genes regulating blood glucose were up-regulated in fast grow fish group in liver tissue [11]. In this study, we found *UGP2*b gene, which was an important intermediary in carbohydrate interconversions was up-regulated in H group. *UGP2* gene was also found to have significant dominant effects on body weight in giant freshwater prawn [14]. Moreover, we found many genes related to lipid metabolism were differentially expressed in previous studies, and we also observed this pattern in liver tissue in this study. *APOBA* gene responsible for lipoprotein synthesis was among the 204 DEGs, and it had been found to have distinct expression levels in pig liver tissue with different growth rates [27].

Identification of genes related with quantitative traits is hard using only one approach due to complex inheritance of these traits and limited resolving power given by the individual techniques. In this study, we found many DEGs in the two tissues were within QTL regions in previous study. In pig, *slc1a7* was found to be significantly differentially expressed in different dietary situations [29]. Knockdown of *ABCF2* using specific siRNA notably increased apoptosis in human [3]. In our previous study in F1 family, we identified a large number of QTLs for growth related traits, including body weight (BW), body length (BL), total length (TL), body height (BH) and head length (HL) [10]. We also found these traits were positively correlated with body weight, which was the phenotype used in this study. Therefore, we investigated the potential association among DEGs overlapped with those QTLs for their influence on growth trait. A deeper analysis of these 9 DEGs within QTL revealed 3 genes were functionally linked to the trait of interest. The *FUK* gene codes for an enzyme which catalyzes the first step in the utilization of free L-fucose in glycoprotein and glycolipid synthesis [31], *ABCF2* gene is associated with transportation of various molecules across extra- and intracellular membranes [1] and *PLA1A* gene encodes a phospholipase that hydrolyzes fatty acids at the sn-1 position of phosphatidylserine and 1-acyl-2-lysophosphatidylserine [2].

## Conclusions

We used RNA-Seq as a tool to explore the functions of hypothalamus-pituitary-liver axis in growth regulation in bighead carp. The differential expression analyses performed by HISAT2-Stringtie-Ballgown revealed 173 and 204 DEGs in HP tissue and liver between H and L group, respectively. Those genes were enriched in many pathways and gene networks related to fatty acid and sterol metabolism. Among those DEGs, 4 and 5 were located within growth related QTL previously identified in the two tissues, and 3 out of all 9 DEGs were found functionally associated with growth traits. Besides, we also found 2442 lncRNAs expressed in HP and liver tissue, from which 164 were liver-specific expressed and 749 were HP-specific. Above all, the results of the present study greatly contributed to the knowledge about the regulation of growth and can help in the design of new selection strategies to improve the growth rates in bighead carp.

## Materials and methods

### Ethical statement

All the fish used in this study were owned by the authors. All experimental procedures in this study about dealing with the fish were approved by the Committee for Animal Experiments of the Institute of Hydrobiology of the Chinese Academy of Sciences, China. The used methods were carried out in accordance with the Laboratory Animal Management Principles of China.

### Sampling for RNA sequencing

Bighead carp juveniles from one family were reared in one pond of the Zhangdu Lake Fishery Farm in Wuhan, China. To eliminate the influence of growth rates by different sex, we only selected female bighead carp in this study. The sex of these samples was detected by a sex-specific primer [17]. Three samples with largest body weight (BW) were clustered in High group (H) and the BW were 845 g, 758 g and 738 g, respectively. Another three samples with smallest BW were clustered in Low group (L) with BW of 496 g, 483 g and 477 g. All fish were slaughtered on the 550 day after fertilization, and before this, MS222 with 140 ppm was used to anesthesia all the fish The hypothalamus and pituitary tissues were washed with pure water, cut into pieces, mixed together and then preserved in RNA later (Invitrogen, USA) and stored in − 80 °C refrigerator before total RNA extraction. The liver tissue was treated with the same methods as before.

### RNA sequencing and reads mapping

Total RNA extraction was carried out using the Promega Z3100 kit (Promega, USA) with DNase treatment following the manufacturer’s instructions. Both hypothalamus-pituitary samples and liver samples were barcoded and sequenced on Illumina HiSeq2500 platform (150 bp paired-end). Sequencing reads quality was checked using FastQC v0.11.3 (http://www.bioinformatics.babraham.ac.uk/projects/fastqc/). Removal of adaptor sequences and quality filtering was conducted on read pairs with Perl scripts. Only the reads for which both pairs were longer than 60 bp post-filtering were retained. Filtered reads were mapped to bighead carp genome (Shunping He, et al. unpublished data) using HISAT v2.0.0 with default parameters [15]. StringTie v1.2.0 [26] was used to build general feature format (GFF) file for each sample and then we merged all 12 GFF files into one consensus file with default parameters.

### Gene expression quantification and differential gene expressed analysis

Based on the consensus GFF file, StringTie v1.2.0 was used to quantify the expression of transcripts and genes in each sample. After that, table counts needed in Ballgown package [9] for differential gene expression analysis were also created by StringTie. Given one common issue in RNA-Seq data is that some genes often have very few counts, we used an approach that can remove these genes by applying a variance filter. We only kept transcripts or genes with variance larger than 1 between H and L groups in any tissue [25].

### LncRNA identification

To identify lncRNAs in all transcripts, we used stringent criteria that combined coding ability and exon number. First, we mapped all transcripts to zebrafish proteome and discarded transcripts with positive results. Second, we checked the exon number for all kept transcripts whether they have more than two exons. Those transcripts which matched these criteria were sent to CPAT v1.2.2 to judge their coding probability. Only transcripts with coding probability less than 0.3 were kept as true lncRNAs.

## Additional files

Additional file 1: **Table S1.** KEGG enrichment analysis of highly expressed genes in liver tissue. (XLSX 13 kb)

Additional file 2: **Table S2.** List of 50 top highly expressed genes in liver tissue. (XLSX 12 kb)

Additional file 3: **Table S3.** GO enrichment analysis of 430 hightly expressed genes in HP tissues. HP: Hypothalamus-Pituitary. (XLSX 17 kb)

Additional file 4: **Table S4.** Expression level for 574 genes with top expression level in liver tissue. (XLSX 91 kb)

## Abbreviations

DEGs: Differentially expressed genes
FDR: False discovery rate
FPKM: Fragments Per Kilobase of transcript per Million mapped reads
GFF: General Feature Format
GH: Growth hormone
GO: Gene ontology
HP: Hypothalamus-pituitary
KEGG: Kyoto Encyclopedia of Genes and Genomes
lncRNA: Long non-coding RNA
QTL: Quantitative trait locus
RNA-Seq: RNA sequencing
